# Multilevel Analysis of Body Composition in Elite and Sub-Elite Female Volleyball Players: Structural and Potentially Modifiable Characteristics

**DOI:** 10.3390/sports14060223

**Published:** 2026-05-29

**Authors:** Matteo Pincella, Fabrizio Spataro, Anjumol Cancian, Alberto Cecchinato, Emanuela Longa, Federica Sprenger, Giuseppe Annunziata, Giuseppe Cerullo, Francesco Campa

**Affiliations:** 1 FIGC Federazione Italiana Giuoco Calcio (Italian Football Federation), 00198 Rome, Italy; studiopincella@gmail.com; 2 PhD School of Applied Medical-Surgical Sciences, Tor Vergata University of Rome, Via Montpellier 1, 00133 Rome, Italy; fabrizio.spataro@gmail.com; 3 Department of Biomedical Sciences, University of Padua, 35131 Padua, Italy; maryanju.cancian@gmail.com (A.C.); alberto.cecchinato.2@studenti.unipd.it (A.C.); giuseppe.cerullo@unipd.it (G.C.); 4 FIPAV, Federazione Italiana Pallavolo (Italian Volleyball Federation), 00189 Rome, Italy; emanuela.longa@unipv.it (E.L.); nutrizionistasprenger@gmail.com (F.S.); 5 Department for the Promotion of Human Sciences and Quality of Life, San Raffaele Open University, 00166 Rome, Italy; giuseppe.annunziata@unina.it

**Keywords:** anthropometry, body proportions, fat mass index, skeletal muscle mass, somatotype

## Abstract

Body composition is a key factor in volleyball performance, but research on female athletes has largely focused on only a few general traits. This study compared elite and sub-elite female volleyball players using a multilevel body composition framework to compare structural and potentially modifiable characteristics across competitive levels. Forty female volleyball players were assessed and classified as elite (n = 15) or sub-elite (n = 25). Body composition was assessed using anthropometry and ultrasound. Elite players were taller (183.1 ± 8.2 vs. 170.7 ± 8.8 cm), heavier (76.0 ± 8.5 vs. 65.8 ± 9.1 kg), and displayed distinct body proportions compared with sub-elite players. The elite group also showed higher skeletal muscle index (SMI: 8.1 ± 0.7 vs. 7.3 ± 0.7 kg·m^−2^) and lower fat mass percentage (22.3 ± 2.2 vs. 25.3 ± 4.4%). However, differences in adiposity were attenuated when normalized for stature using fat mass index (FMI: 5.1 ± 0.8 vs. 5.8 ± 1.5 kg·m^−2^). Ultrasound-derived data indicated greater regional muscularity in elite players, whereas no differences were observed in the sum of adipose tissue layers, consistent with anthropometric skinfolds. The muscle-to-bone ratio did not differ between groups, suggesting proportional development of muscle and bone mass. Elite female volleyball players were characterized by greater structural dimensions and muscularity, whereas FMI appeared more informative than FM% for assessing adiposity.

## 1. Introduction

Volleyball is a high-intensity intermittent team sport characterized by repeated explosive actions such as jumping, blocking, spiking, and rapid changes in direction [1]. Performance in volleyball depends on the interaction of technical, tactical, physical, and morphological factors, with body structure playing a particularly important role in net-related actions where reach, leverage, and force production may directly influence competitive success [2].

**Figure 1:**
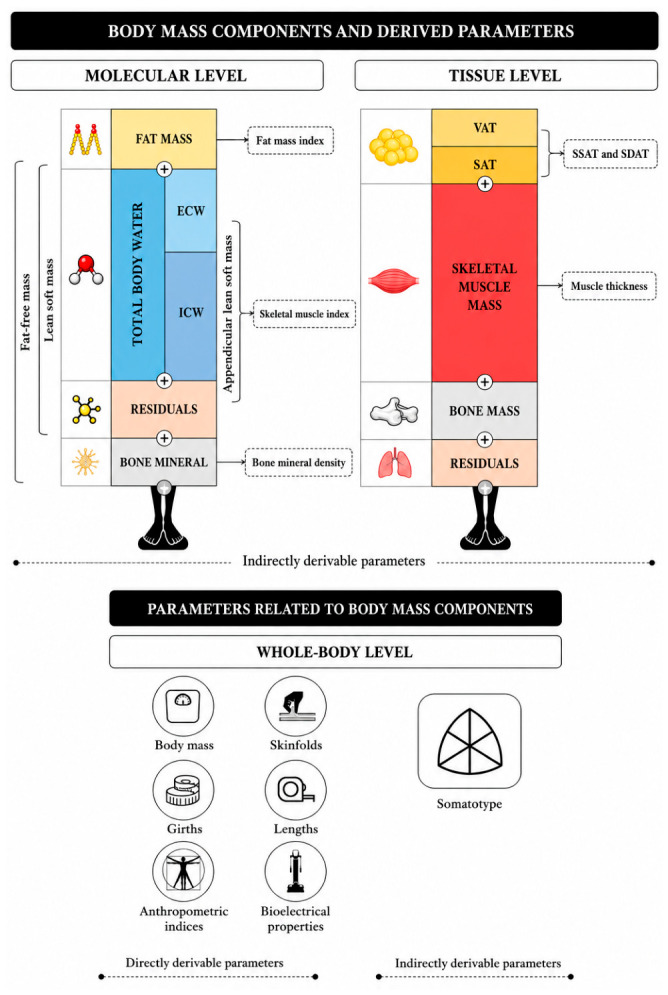
Multilevel organizational framework of body composition, illustrating molecular, tissue, and whole-body levels. ECW, extracellular water; ICW, intracellular water; SAT, subcutaneous adipose tissue; SSAT, superficial subcutaneous adipose tissue; DSAT, deep subcutaneous adipose tissue; VAT, visceral adipose tissue. Adapted from Wang et al. [5].

Among the factors associated with volleyball performance, body composition has long been recognized as relevant for both athlete profiling and long-term athlete development [1]. Traditionally, body composition has often been examined in terms of body mass components such as fat mass (FM) and skeletal muscle mass (SMM) [3]. However, body composition can also be interpreted within a multilevel organizational framework, in which the human body is described across different levels of biological organization [4], ranging from body mass components to whole-body descriptors such as size, shape, and proportionality, as shown in Figure 1. Within this perspective, anthropometric dimensions, segment lengths, skeletal breadths, girths, proportional indices, and somatotype are not separate from body composition, but rather represent complementary expressions of the athlete’s morphological characteristics.

In volleyball, this multilevel perspective may be particularly relevant. Certain body traits, such as greater stature, longer limb segments, and broader skeletal dimensions [6], may be associated with mechanical advantages in blocking, attacking, and vertical reach [2,7,8]. In parallel, body composition variables such as lower adiposity and greater muscular development may contribute to explosive performance, repeated jumping ability, and overall physical efficiency during match play [4,9]. These characteristics may contribute to the descriptive morphological profile observed in high-level volleyball players [10].

An additional practical implication of this approach may lie in distinguishing traits that are potentially modifiable from those that are generally less modifiable during adulthood [6]. Variables related to adiposity and muscularity may be more responsive to training and nutritional interventions [1,10], whereas skeletal dimensions, body lengths, and proportional indices are generally considered less modifiable characteristics [11]. Consequently, the simultaneous assessment of these traits may provide useful information for athlete profiling and for the interpretation of training- and nutrition-related adaptations in volleyball players.

Despite the recognized importance of body composition in volleyball, the available literature remains relatively limited in female athletes [12]. Most previous studies have focused on a restricted number of general characteristics, such as height, body mass, or body fat percentage, whereas more comprehensive anthropometric profiling, including body proportions, segmental dimensions, skeletal breadths, and somatotype, have been less frequently explored in women’s volleyball [7,13]. This represents an important gap, as a more integrated morphological characterization may improve the understanding of the structural features associated with the competitive level [6]. To our knowledge, no studies have adopted a comprehensive multilevel body composition approach to characterize female volleyball players across competitive levels.

Therefore, the aim of this study was to examine body composition in female volleyball players from a multilevel organizational perspective. Specifically, we compared a wide range of body composition variables with emphasis on structural and potentially modifiable characteristics across competitive levels.

## 2. Materials and Methods

### 2.1. Study Design and Participants

This cross-sectional study aimed to compare body composition between elite and sub-elite female volleyball players using a multilevel organizational approach.

A total of 40 female volleyball players were recruited and classified according to competitive level into two groups: elite (n = 15) and sub-elite (n = 25). Competitive level classification was based on the league in which athletes were actively competing at the time of data collection. The elite group consisted of athletes competing in the Italian Serie A1/A2 championship who had also been selected at least once for a national team program. The sub-elite group consisted of athletes competing in lower national-level Italian championships (i.e., Serie B and lower divisions).

All measurements were performed during the competitive season under standardized conditions. Participants were assessed in a rested state and were instructed to avoid strenuous physical activity prior to testing. Hydration status, nutritional intake, menstrual-cycle phase, and recent training load were not formally recorded or standardized. Before participation, all athletes were informed about the study procedures and provided written informed consent. The study was conducted in accordance with the principles of the Declaration of Helsinki and was approved by the ethics committee of the University of Padua (approval code: HEC-DSB02.2023).

### 2.2. Anthropometric Assessment

Anthropometric assessments were conducted by Level 2 and 3 operators certified by the International Society for the Advancement of Kinanthropometry (ISAK), following standardized international procedures [14]. ISAK certification requires verification of intra- and inter-tester measurement precision according to internationally standardized criteria. Intra-operator Technical Error of Measurement (TEM) values were below 5% for skinfold thicknesses and below 1% for all other anthropometric measurements. Body mass and stature were measured using a scale with an integrated stadiometer (Seca, Hamburg, Germany) to the nearest 0.1 kg and 0.1 cm, respectively. Skinfold thicknesses were measured using a type A [15] skinfold caliper (Holway Tools, Los Angeles, CA, USA) at eight anatomical sites: triceps, subscapular, biceps, iliac crest, supraspinale, abdominal, thigh, and calf, and their sum was calculated. Girth measurements were obtained using a non-elastic anthropometric tape (Lufkin, Apex Tool Group, Soarks, MD, USA) with a sensitivity of 0.1 mm, including head, neck, arm relaxed, flexed and tensed arm, forearm, wrist, chest, waist, hips, thigh 1 cm gluteal, thigh middle, calf, and ankle. Breadths and segment lengths were measured to the nearest 0.1 cm using a sliding caliper (Holway, Tools, Los Angeles, CA, USA). These included biacromial, biiliocristal, chest, humerus, femur, bi-styloid, and bimalleolar breadths, as well as multiple segment lengths (upper and lower limbs and trunk-related measures). Somatotype was determined according to the Heath–Carter anthropometric method [16].

Several anthropometric proportional indices were derived from the linear and breadth measurements to characterize segmental proportions and overall body shape. The relative arm span was calculated as arm span divided by stature and multiplied by 100, whereas the Cormic index was calculated as sitting height divided by stature and multiplied by 100. These indices were used to describe upper-limb reach and trunk proportionality relative to total body height, respectively. The brachial index was calculated as radiale–stylion length divided by acromiale–radiale length and multiplied by 100, thereby expressing forearm length relative to upper arm length. The intermembral index was calculated as total upper-limb length divided by total lower-limb length and multiplied by 100, where total upper-limb length was defined as the sum of acromiale–radiale, radiale–stylion, and midstylion–dactylion lengths, and total lower-limb length as the iliospinale height. The crural index was calculated as tibiale mediale–sphyrion tibiale length divided by trochanterion–tibiale laterale length and multiplied by 100, representing lower leg length relative to thigh length. Finally, the acromio-iliac index was calculated as biiliocristal breadth divided by biacromial breadth and multiplied by 100, and was used to describe shoulder-to-pelvis proportionality. All indices were expressed as percentages.

The muscle-to-bone ratio was estimated using anthropometric prediction equations based on the Kerr fractionation model [17], based on the proportionality system relative to the Phantom reference developed by Drinkwater and Ross [18]. For each tissue compartment (muscle and bone), a Z-score was calculated using the equation Z = [(V × (170.18/H)) − P]/s, where V is the sum of the compartment-specific anthropometric variables, H is stature (cm), P is the Phantom reference value, and s is the corresponding Phantom standard deviation. In the equation M = (Z × s + P) × (H/170.18)^3^, M represents the estimated tissue mass expressed in kilograms, Z is the standardized Phantom Z-score, s is the Phantom standard deviation, P is the Phantom reference value, and H is stature in centimeters. Muscle mass was estimated from the sum of corrected girths (arm, forearm, chest, thigh, and calf), adjusted for subcutaneous adipose tissue using the equation Corrected girths = C − π × (SKF/10), where C is girth (cm), SKF is the corresponding skinfold thickness in mm, and π is the mathematical constant pi. Bone mass was estimated by combining the contribution of the body (based on biacromial and biiliocristal breadths, and humerus and femur bicondylar breadths) and the head (derived from head girth), following the original Kerr formulation. Tissue masses were calculated independently of total body mass, and no post hoc adjustment was applied to match measured body mass. Higher muscle-to-bone ratio values indicate relatively greater estimated muscle mass in relation to estimated bone mass.

### 2.3. Ultrasound Assessment

**Figure 2:**
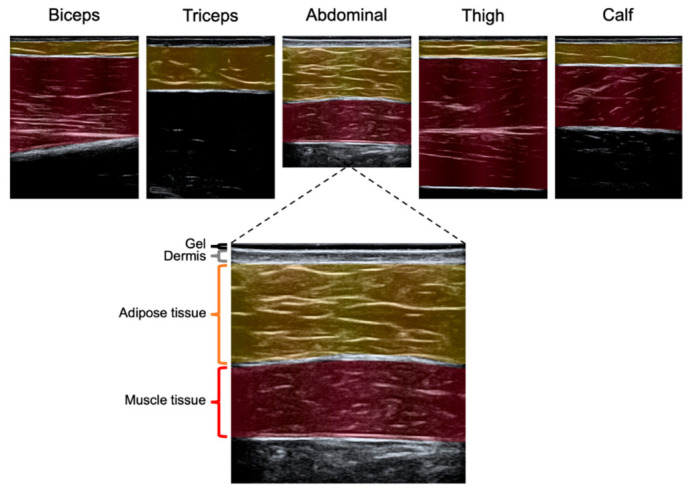
Representative B-mode ultrasound images acquired at the anatomical sites assessed in this study (biceps, triceps, abdominal, thigh, and calf). The images illustrate the layered structure of superficial tissues, including the gel/dermis interface, subcutaneous adipose tissue (yellow), and underlying skeletal muscle (red). Adipose tissue was identified at all sites, whereas muscle thickness was not assessed at the triceps due to anatomical and methodological constraints [19]. The abdominal panel provides a detailed example of tissue stratification, highlighting the clear distinction between subcutaneous adipose and muscle layers used for thickness measurements.

Regional adipose and muscle thickness was assessed using a wireless B-mode ultrasound device (Ultracomp One, Hitasonix, Milan, Italy). Ultrasound images were acquired at predefined anatomical landmarks according to the standardized procedures recommended by the manufacturer and described in a previous study on male and female adults [19]. According to the standardized acquisition procedures, subcutaneous adipose tissue thickness was identified and measured at all five anatomical sites (biceps, triceps, abdomen, thigh, and calf). Muscle thickness was assessed at all sites except the triceps [19]. Specifically, arm sites corresponded to the triceps and biceps, located at the mid-posterior and mid-anterior portions of the upper arm, respectively, with the probe positioned longitudinally over the same landmarks used for skinfold assessment. The abdominal site was located 5 cm lateral to the umbilical center, with the probe placed transversely. The thigh site was measured at mid-thigh level with the participant seated on an anthropometric box and the knee fully extended, positioning the probe longitudinally over the standard anthropometric landmark. The calf measurement was taken at the medial calf site, with the leg flexed and supported on a box, placing the probe longitudinally along the corresponding anthropometric point. This protocol was intentionally designed to align ultrasound measurements with conventional anthropometric landmarks, thereby ensuring comparability across assessment methods [19]. Figure 2 illustrates representative ultrasound images and highlights the typical stratification of superficial tissues, with the abdominal site providing a clear example of layer differentiation used for measurement.

Measurements were performed by a trained operator with excellent reliability (ICC = 0.997). Measurement repeatability was evaluated through a test–retest procedure conducted in 20 subjects, with all scans performed twice by the same operator under standardized conditions. Based on these repeated assessments, the minimum detectable change at the 95% confidence level (MDC95) was calculated for each ultrasound-derived variable. MDC95 values were 0.70 mm for biceps fat, 3.3 mm for biceps muscle, 0.70 mm for triceps fat, 1.3 mm for abdominal fat, 1.9 mm for abdominal muscle, 0.60 mm for thigh fat, 2.5 mm for thigh muscle, 1.5 mm for calf fat, and 4.5 mm for calf muscle. The MDC95 was calculated as: MDC95 = SEM × 1.96 × √2. The identified anatomical reference sites were entered into the dedicated device software (Ultracomp One, Hitasonix, Milan, Italy), which was used to estimate FM and SMM based on the regional tissue thickness measurements obtained from the ultrasound scans. To account for body size, fat mass index (FMI) and skeletal muscle index (SMI) were calculated as commonly used indices to normalize body composition variables for stature. FMI was calculated as FM divided by stature squared (kg·m^−2^), and SMI as SMM divided by stature squared (kg·m^−2^).

### 2.4. Statistical Analysis

Statistical analyses were performed using SPSS version 31 (IBM Corp., Armonk, NY, USA). Descriptive statistics are reported as mean ± standard deviation (SD). Data distribution was assessed using the Shapiro–Wilk test. Homogeneity of variances was evaluated using Levene’s test. Because several variables violated the assumption of equal variances between groups, Welch’s *t*-test was used for between-group comparisons. Effect sizes were calculated using Hedges’ g together with 95% confidence intervals. To control for the risk of type I error associated with multiple comparisons, *p*-values were adjusted using the Benjamini–Hochberg false discovery rate (FDR) procedure, with statistical significance set at q < 0.05 after correction. Positional analyses were limited to descriptive statistics only, as the number of athletes within each playing position subgroup was insufficient to support reliable inferential comparisons.

## 3. Results

The elite and sub-elite groups had a mean age of 25.1 ± 4.9 and 22.0 ± 6.2 years, respectively (*p* = 0.134). Descriptive statistics and between-group comparisons for structural and proportional traits and potentially modifiable traits are reported in Table 1 and Table 2, respectively.

**Figure 3:**
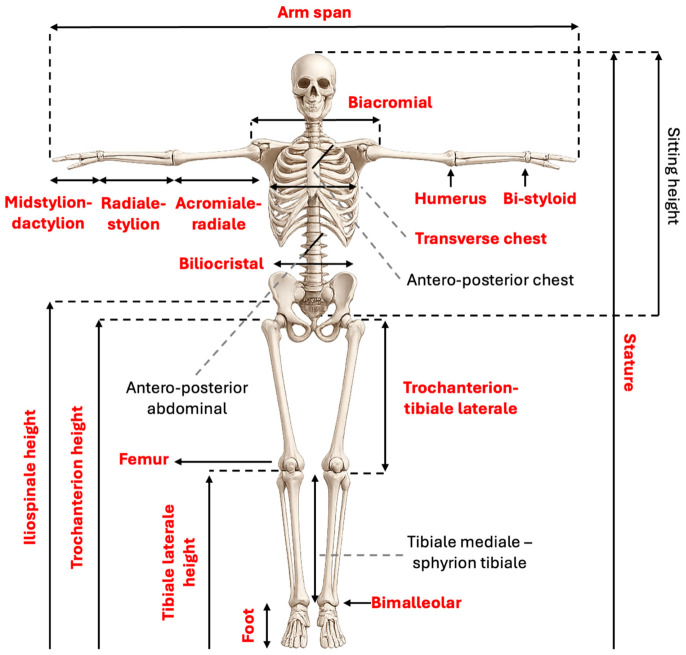
Illustration of lengths, heights, breadths, and depths. Red labels indicate variables with significantly higher mean values in elite players after FDR correction. No parameters were significantly higher in the sub-elite group.

The elite athletes were significantly taller and displayed greater arm span, broader skeletal dimensions, and longer upper- and lower-limb segments (Table 1). They also showed greater body mass and larger body girths across several anatomical sites (Table 2). At the proportional level, elite players exhibited a significantly lower cormic index and crural index. Figure 3 shows the skeletal parameters, descriptively illustrating the differences between the two groups.

Considering body mass components, the elite players presented significantly higher SMM and SMI, bone mass, and several ultrasound-derived muscle thickness variables (Table 2).

**Figure 4:**
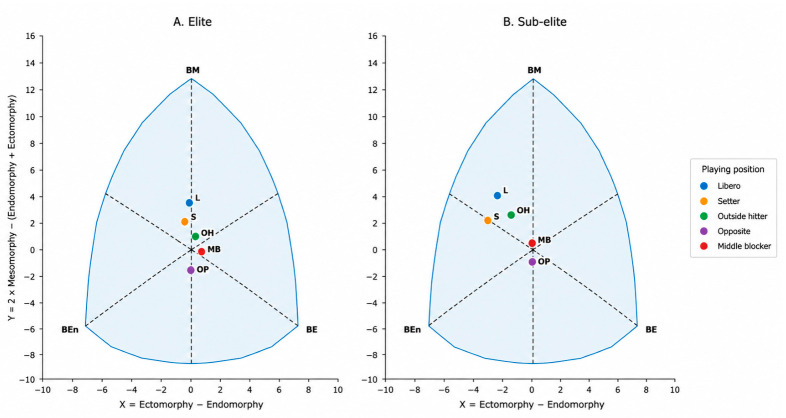
Somatotype distribution of the female volleyball players by playing position and competitive level. Panel (**A**) shows elite players, and panel (**B**) shows sub-elite players.

Descriptive statistics stratified by playing position are reported in the Supplementary Tables (Supplementary Tables S1–S5). The positional distribution of the elite and sub-elite groups was relatively comparable across roles. Specifically, the elite group included two liberos, three setters, five outside hitters, three opposite hitters, and two middle blockers, whereas the sub-elite group included four liberos, five setters, five outside hitters, six opposite hitters, and five middle blockers. Because of the limited number of athletes available within each positional subgroup, only descriptive statistics were reported, and no formal inferential comparisons were performed by playing position. Somatotype values by playing position and competitive level are reported in Table 3, and their distribution is illustrated in Figure 4.

## 4. Discussion

The present study examined body composition in female volleyball players using a multilevel organizational approach, with the aim of characterizing different competitive levels and descriptively examining structural and potentially modifiable characteristics. Overall, the findings indicate that elite female volleyball players display a distinct morphological profile characterized by greater body size, broader skeletal dimensions, distinct body proportions, and greater muscularity compared with sub-elite athletes.

These results align with the multilevel perspective of body composition, which emphasizes the interaction between structural and functional components of the human body in characterizing sport-specific profiles [4,20]. Moreover, they extend previous findings in the volleyball literature by providing a more comprehensive characterization of female athletes, in whom integrated morphological profiling has been less extensively investigated [1,7,12]. One of the most relevant findings of the present study is that elite players differed from sub-elite players not only in absolute body size, but also in body proportions. In particular, elite athletes exhibited a lower cormic index and lower crural index, indicating relatively longer lower limbs in relation to trunk length. From a biomechanical perspective, these proportions are highly relevant in volleyball, as longer lower limbs may contribute to greater standing reach capacity, more favorable conditions for vertical actions, and potential mechanical advantages [8,21]. These findings are consistent with previous studies reporting the importance of limb proportions and segment lengths in volleyball performance [2,6], although direct comparisons with female cohorts remain limited. Indeed, it has been reported that higher-level volleyball players and front-row positions tend to exhibit greater stature and longer segmental dimensions, characteristics considered advantageous for blocking and attacking actions [8,21]. Taken together, the present findings suggest that body proportionality, particularly lower cormic index values and relatively longer lower limbs, may characterize the morphological profile observed in the elite players.

Elite athletes also showed greater skeletal robustness, as reflected by broader biacromial breadth and other transverse skeletal dimensions. A broader shoulder girdle may provide both mechanical and functional advantages in volleyball, particularly in actions requiring force transmission through the upper body such as serving, spiking, and blocking. This observation is in agreement with previous anthropometric studies highlighting the importance of skeletal breadths in high-level volleyball players [7,13]. Importantly, stature and skeletal breadths may be considered relatively less modifiable structural traits during adulthood. In this context, taller athletes with broader skeletal dimensions may be associated with greater absolute SMM values, both from a biomechanical and physiological perspective [22]. Therefore, these structural characteristics may contribute to the morphological profile observed in higher-level volleyball players, supporting their potential relevance within athlete profiling frameworks.

From the perspective of potentially modifiable traits, the elite players were characterized by greater muscular development. This was consistently observed across estimated body mass components and ultrasound-derived regional measurements. Elite athletes showed higher SMM and SMI, as well as greater muscle thickness at several anatomical sites. This observation aligns with previous research linking muscularity to performance-relevant actions in volleyball, particularly jumping and hitting [6,9]. From an applied perspective, this finding is particularly meaningful, as muscular development is generally considered more responsive to training interventions, and previous studies in elite female volleyball players have shown that body composition can vary across the competitive season [10]. In volleyball, greater muscularity may support repeated explosive actions, including jumping, landing, and rapid changes in direction, and may therefore represent a relevant descriptive characteristic of higher-level athletes [23,24]. The FM percentage of the elite players in the present study is also closely aligned with the pooled estimate reported in the recent systematic review and meta-analysis [1], which identified an average body fat value of 22.8%, ranging from 21.9 to 23.7%, in female volleyball players. Interestingly, although elite players showed lower FM%, this between-group difference was attenuated when adiposity was normalized for stature using FMI, suggesting that conventional percentage-based indicators may overestimate adiposity differences in samples characterized by substantial stature variation. This consideration is particularly relevant in volleyball, where large differences in stature are common and may confound the interpretation of conventional body composition indicators [12]. Therefore, the present findings support the use of height-adjusted indices, particularly FMI and SMI, when evaluating body composition in female volleyball players.

Ultrasound-derived data provided further insight into the tissue-level characteristics of the two groups. The elite players showed greater regional muscularity, whereas no significant differences were observed in the sum of adipose tissue layers, in agreement with the lack of significant differences in the global anthropometric sum of skinfolds. This pattern partially agrees with previous studies suggesting that differences in performance level may be more strongly related to muscle characteristics than to subcutaneous fat distribution [9]. This observation suggests that the primary compositional distinction between elite and sub-elite players lies more in muscle development than in subcutaneous adiposity per se. Elite athletes appeared to be characterized more by greater muscularity than by lower subcutaneous adiposity alone. This distinction may have practical implications, as it shifts the focus from generalized fat reduction toward the development and maintenance of sport-specific muscularity. Although ultrasound-derived body composition data in female volleyball players are still scarce, previous studies in other athletic populations have similarly shown that regional muscle thickness may provide relevant information regarding sport-specific muscular development and tissue characteristics [25].

**Figure 5:**
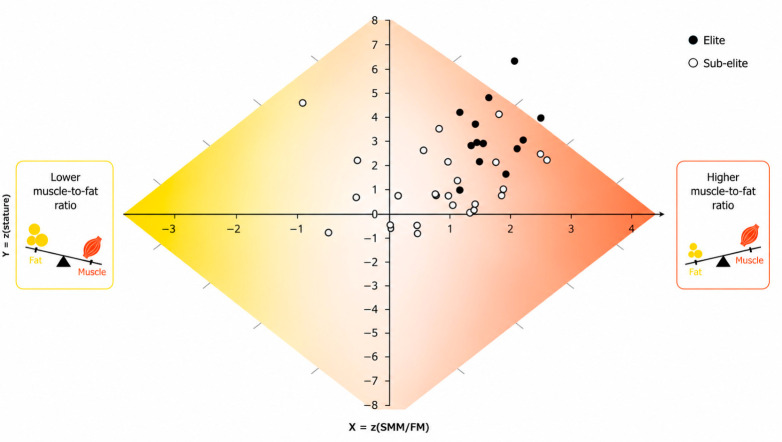
Standardized body composition map based on stature and skeletal muscle mass-to-fat mass. Z-scores were calculated using sex-specific general population values [26,27] (males: fat mass-to-skeletal muscle mass ratio = 0.55 ± 0.22, stature 176.99 ± 7.10 cm; females: fat mass-to-skeletal muscle mass ratio = 0.98 ± 0.33, stature 163.28 ± 6.00 cm). Black circles indicate elite players, and white circles indicate sub-elite players.

To further contextualize the findings in relation to body mass components, their proportional relationships, and skeletal characteristics, players were also represented using a standardized bivariate model based on sex-specific reference values from the general population [26,27] (Figure 5). Specifically, z-scores were calculated for stature and the FM/SMM ratio using the following reference values: for males, FM/SMM = 0.55 ± 0.22 and stature = 176.99 ± 7.10 cm; for females, FM/SMM = 0.98 ± 0.33 and stature = 163.28 ± 6.00 cm. This approach allowed each athlete to be positioned relative to sex-specific population standards, thereby providing an integrated visualization of body frame and relative tissue composition. Since these reference values were derived from a general population sample rather than athletic cohorts, the resulting map should be interpreted as a descriptive comparative tool, and not as a sport-specific normative reference. Within this framework, elite players (black dots) appeared descriptively distributed toward lower FM/SMM ratios and greater stature values compared with sub-elite players (white dots). Importantly, within this descriptive framework, variation between groups appeared more evident along the axis representing body mass component relationships.

An additional methodological consideration concerns the distinction between ultrasound-derived SMM and anthropometrically estimated muscle mass. Specifically, SMM was derived from equations developed against DXA-derived appendicular lean soft mass [19] and therefore converted at the tissue level using a validated approach [28], whereas the Kerr model [17] estimates a broader muscle compartment and is based on cadaver dissection data. As a result, these variables should not be interpreted interchangeably, and different equations may yield different estimates depending on the reference method and population in which they were developed. Data on SMM in female volleyball players are still scarce, which limits direct comparisons with previous studies. Finally, to our knowledge, this is the first study to apply ultrasound for body composition assessment in female volleyball players. This is relevant from an applied perspective, as ultrasound may represent a practical and non-invasive tool that can be used across different professionals within the same support staff, extending its utility beyond rehabilitation settings [29,30].

Another interesting finding is that the muscle-to-bone ratio did not differ between elite and sub-elite players. Despite elite athletes having significantly greater estimated muscle mass and bone mass, these components appeared to increase proportionally. This suggests that the elite group was not characterized by disproportionate muscularity relative to skeletal structure, but rather by a more globally developed musculoskeletal framework [4]. From a biological and functional perspective, this is plausible, as previous studies in female volleyball players have shown greater bone and soft-tissue development in association with sport-specific loading [31,32]. This finding also supports the interpretation of elite volleyball players as exhibiting greater musculoskeletal development while maintaining proportionality between tissue compartments.

The descriptive somatotype analysis further complements this interpretation. Although inferential comparisons were not performed due to the limited number of athletes within each positional subgroup, the elite players generally appeared more frequently distributed within the mesomorphic–ectomorphic region, whereas the sub-elite athletes showed a more endomorphic and dispersed distribution. This pattern is consistent with previous studies reporting mesomorphic predominance in high-level volleyball players [13,33]. In particular, Martín-Matillas et al. [13] and Malousaris et al. [33] described elite female volleyball players as predominantly mesomorphic–ectomorphic, especially in positions requiring repeated jumping and net-related actions. Accordingly, somatotype should be interpreted here as a descriptive summary of body shape rather than an explanatory factor for playing a role, particularly given the limited sample size within positional subgroups.

Taken together, the present findings may have practical implications for athlete profiling and long-term athlete development in female volleyball. Structural traits such as stature, skeletal breadths, and body proportions may contribute to the characterization of volleyball-specific morphological profiles, whereas more modifiable traits related to muscularity and adiposity may represent variables of interest for training and nutritional monitoring. However, because of the cross-sectional nature of the study, these characteristics should not be interpreted as causal determinants of elite performance. Rather, the present findings should be interpreted as characteristics observed across competitive levels that may help inform future longitudinal research and athlete monitoring approaches.

Some limitations of this study should be acknowledged. First, the sample size was relatively modest, particularly when participants were stratified by playing position, which limited positional analyses to descriptive statistics. This aspect may have partially confounded the observed differences between elite and sub-elite players, since volleyball playing positions are characterized by distinct anthropometric and body composition profiles [6]. Second, the cross-sectional design does not allow causal inferences regarding whether the identified characteristics are determinants or consequences of elite performance. Another limitation concerns the estimation procedures used for ultrasound-derived body composition variables. Although the software-derived estimates of FM and SMM were developed and previously described in a physically active adult population [19], further validation studies in athletic populations, particularly female volleyball players, are still warranted. However, the primary aim of the present study was not to provide normative reference values, but rather to compare raw and relative body composition characteristics between groups within the same methodological framework. Therefore, even if some systematic estimation bias were present, it would be expected to affect both groups similarly and would not substantially alter the interpretation of the between-group comparisons. Lastly, hydration status, nutritional intake, menstrual-cycle phase, and recent training load were not formally standardized, which may have contributed to variability in body composition measurements [34]. Nevertheless, a major strength of the present study lies in the integration of anthropometry, derived body composition indices, ultrasound-derived tissue measurements, and somatotype within a single analytical framework, providing a more comprehensive characterization of female volleyball players than is typically reported in the literature.

## 5. Conclusions

In conclusion, elite female volleyball players were characterized by greater structural dimensions, distinct body proportions, and greater muscularity compared with sub-elite athletes within a multilevel body composition framework. These findings support the usefulness of descriptively considering both relatively structural characteristics and potentially modifiable body composition variables when profiling female volleyball players. Given the cross-sectional design and limited sample size, the findings should be interpreted as descriptive differences between competitive levels rather than causal or predictive indicators of elite performance.

## Figures and Tables

**Table 1:** Structural and proportional traits of the elite and sub-elite female volleyball players.

Variable	Elite	Sub-Elite	*t* (Welch)	*p* (FDR)	Hedges’ g (95% CI)
Stature (cm)	183.07 ± 8.16	170.72 ± 8.78	4.504	0.001	1.42 (0.70 to 2.13)
Sitting height (cm)	90.27 ± 3.81	90.17 ± 3.50	0.084	0.934	0.03 (−0.61 to 0.67)
Arm span (cm)	186.93 ± 11.24	173.62 ± 10.17	3.756	0.003	1.23 (0.54 to 1.93)
Girths					
Head girth (cm)	56.76 ± 2.46	54.98 ± 1.68	2.486	0.038	0.87 (0.20 to 1.54)
Wrist girth (cm)	16.21 ± 0.58	15.48 ± 0.61	3.455	0.001	1.03 (0.35 to 1.72)
Ankle girth (cm)	23.47 ± 1.45	21.89 ± 1.27	3.480	0.005	1.15 (0.46 to 1.84)
Proportional indices					
Acromio-iliac index (%)	73.01 ± 3.59	75.11 ± 6.84	−1.274	0.258	−0.35 (−1.00 to 0.29)
Relative arm span (%)	102.07 ± 3.13	101.72 ± 3.89	0.306	0.795	0.09 (−0.55 to 0.73)
Brachial index (%)	78.14 ± 3.77	79.23 ± 11.19	−0.443	0.721	−0.12 (−0.76 to 0.53)
Cormic index (%)	49.35 ± 1.80	52.87 ± 1.57	−6.270	<0.001	−2.08 (−2.87 to −1.29)
Crural index (%)	82.44 ± 6.09	86.74 ± 2.83	−2.574	0.036	−0.97 (−1.65 to −0.30)
Intermembral index (%)	79.25 ± 2.77	77.90 ± 3.01	1.437	0.211	0.45 (−0.20 to 1.10)
Heights and lengths					
Acromiale–radiale length (cm)	35.33 ± 2.40	32.06 ± 3.26	3.641	0.003	1.08 (0.40 to 1.76)
Radiale-stylion length (cm)	27.57 ± 1.75	25.13 ± 1.82	4.216	0.001	1.34 (0.63 to 2.04)
Midstylion–dactylion length (cm)	20.44 ± 1.41	18.62 ± 1.12	4.239	0.002	1.44 (0.72 to 2.16)
Iliospinale height (cm)	105.27 ± 7.12	97.35 ± 5.75	3.655	0.004	1.23 (0.54 to 1.93)
Trochanterion height (cm)	100.04 ± 7.32	91.73 ± 5.58	3.782	0.001	1.31 (0.60 to 2.)
Trochanterion–tibiale laterale length (cm)	50.25 ± 3.94	45.63 ± 3.19	3.839	0.003	1.30 (0.59 to 2.00)
Tibiale laterale height (cm)	50.34 ± 3.64	45.69 ± 2.75	4.263	0.002	1.46 (0.74 to 2.18)
Tibiale mediale–sphyrion tibiale length (cm)	41.39 ± 4.09	39.55 ± 2.53	1.577	0.178	0.57 (−0.09 to 1.22)
Foot length (cm)	26.85 ± 1.29	25.00 ± 1.50	4.140	0.001	1.28 (0.57 to 1.98)
Bone-related variables					
Biacromial breadth (cm)	40.37 ± 2.43	36.90 ± 2.77	4.155	0.001	1.29 (0.58 to 1.99)
Biiliocristal breadth (cm)	29.43 ± 1.42	27.62 ± 2.13	3.217	0.007	0.93 (0.26 to 1.61)
Transverse chest breadth (cm)	28.99 ± 1.48	26.52 ± 1.49	5.103	<0.001	1.63 (0.89 to 2.37)
Antero-posterior chest depth (cm)	16.35 ± 1.05	16.06 ± 1.02	0.853	0.459	0.27 (−0.37 to 0.92)
Antero-posterior abdomen depth (cm)	17.80 ± 1.85	18.50 ± 1.57	−1.223	0.280	−0.41 (−1.06 to 0.24)
Humerus breadth (cm)	6.73 ± 0.23	6.38 ± 0.36	3.701	0.003	1.06 (0.38 to 1.74)
Bi-styloid breadth (cm)	5.45 ± 0.22	5.14 ± 0.30	3.757	0.003	1.12 (0.43 to 1.80)
Femur breadth (cm)	9.58 ± 0.38	9.18 ± 0.40	3.152	0.009	0.99 (0.32 to 1.67)
Bimalleolar breadth (cm)	7.13 ± 0.30	6.85 ± 0.37	2.570	0.031	0.78 (0.12 to 1.44)
Bone mass (kg)	8.00 ± 0.89	6.94 ± 1.05	3.415	0.005	1.05 (0.36 to 1.73)

Note: Values are presented as mean ± standard deviation (SD). Between-group differences were assessed using Welch’s *t*-test. *p*-values were adjusted using the Benjamini–Hochberg false discovery rate (FDR). Hedges’ g values are presented with 95% confidence intervals.

**Table 2:** Potentially modifiable traits of the elite and sub-elite female volleyball players.

Variable	Elite	Sub-Elite	*t* (Welch)	*p* (FDR)	Hedges’ g (95% CI)
Body mass (kg)	75.96 ± 8.53	65.80 ± 9.11	3.556	0.004	1.12 (0.43 to 1.81)
Body mass index (kg·m^−2^)	22.64 ± 1.87	22.54 ± 2.25	0.162	0.898	0.05 (−0.59 to 0.69)
Skinfold thicknesses					
Triceps skinfold (mm)	13.13 ± 3.99	15.45 ± 4.07	−1.763	0.134	−0.56 (−1.21 to 0.09)
Subscapular skinfold (mm)	10.33 ± 2.09	11.34 ± 4.27	−0.996	0.379	−0.27 (−0.92 to 0.37)
Biceps skinfold (mm)	5.37 ± 1.36	6.72 ± 3.22	−1.843	0.119	−0.49 (−1.14 to 0.16)
Iliac crest skinfold (mm)	13.83 ± 3.38	15.18 ± 6.88	−0.829	0.465	−0.23 (−0.87 to 0.42)
Supraspinale skinfold (mm)	8.50 ± 1.94	9.39 ± 3.60	−1.019	0.373	−0.28 (−0.93 to 0.36)
Abdominal skinfold (mm)	14.90 ± 4.07	17.64 ± 6.07	−1.708	0.136	−0.50 (−1.15 to 0.15)
Thigh skinfold (mm)	19.67 ± 4.66	24.34 ± 8.01	−2.335	0.044	−0.66 (−1.32 to 0.00)
Calf skinfold (mm)	10.83 ± 3.57	14.22 ± 4.88	−2.523	0.033	−0.75 (−1.41 to −0.09)
Sum of 8 skinfolds (mm)	96.57 ± 17.01	114.29 ± 36.08	−2.098	0.073	−0.57 (−1.22 to 0.08)
Girths					
Neck girth (cm)	33.97 ± 1.61	33.16 ± 1.63	1.534	0.182	0.49 (−0.16 to 1.14)
Arm relaxed girth (cm)	29.56 ± 1.70	28.03 ± 2.69	2.197	0.059	0.63 (−0.03 to 1.29)
Arm flexed and tensed girth (cm)	30.85 ± 1.90	28.54 ± 2.24	3.477	0.004	1.07 (0.38 to 1.75)
Forearm girth (cm)	25.97 ± 0.82	24.19 ± 1.59	4.673	<0.001	1.29 (0.59 to 1.99)
Chest girth (cm)	93.74 ± 6.27	88.35 ± 5.12	2.814	0.021	0.95 (0.27 to 1.62)
Waist girth (cm)	76.40 ± 4.27	72.33 ± 5.09	2.713	0.022	0.83 (0.16 to 1.50)
Hip girth (cm)	103.29 ± 5.14	98.94 ± 5.54	2.518	0.034	0.79 (0.13 to 1.46)
Thigh 1 cm gluteal (cm)	61.48 ± 3.18	58.13 ± 4.10	2.889	0.016	0.87 (0.20 to 1.54)
Thigh girth (cm)	54.14 ± 3.16	50.98 ± 3.94	2.788	0.020	0.84 (0.18 to 1.51)
Calf girth (cm)	36.99 ± 2.27	35.67 ± 2.33	1.763	0.134	0.56 (−0.09 to 1.21)
Ultrasound-derived layers					
Biceps fat thickness (cm)	0.31 ± 0.12	0.33 ± 0.17	−0.412	0.730	−0.12 (−0.76 to 0.52)
Biceps muscle thickness (cm)	2.59 ± 0.59	2.01 ± 0.30	3.526	0.006	1.32 (0.61 to 2.02)
Triceps fat thickness (cm)	0.77 ± 0.18	0.92 ± 0.39	−1.669	0.144	−0.45 (−1.10 to 0.20)
Abdominal fat thickness (cm)	1.07 ± 0.48	1.15 ± 0.57	−0.513	0.678	−0.16 (−0.80 to 0.48)
Abdominal muscle thickness (cm)	1.35 ± 0.20	1.06 ± 0.18	4.671	0.001	1.54 (0.81 to 2.27)
Thigh fat thickness (cm)	0.75 ± 0.20	0.85 ± 0.25	−1.397	0.217	−0.42 (−1.07 to 0.23)
Thigh muscle thickness (cm)	3.95 ± 0.48	3.70 ± 0.68	1.350	0.231	0.40 (−0.25 to 1.04)
Calf fat thickness (cm)	0.49 ± 0.19	0.60 ± 0.23	−1.713	0.136	−0.52 (−1.17 to 0.13)
Calf muscle thickness (cm)	1.67 ± 0.20	1.50 ± 0.22	2.501	0.034	0.79 (0.12 to 1.45)
Sum of muscle thicknesses (cm)	9.56 ± 0.90	8.27 ± 0.96	4.272	0.001	1.34 (0.64 to 2.05)
Sum of fat thicknesses (cm)	3.39 ± 0.76	3.86 ± 1.37	−1.400	0.217	−0.39 (−1.04 to 0.25)
Body mass components and derived indices					
Fat mass (kg)	16.95 ± 2.79	16.80 ± 4.53	0.128	0.912	0.04 (−0.60 to 0.68)
Fat mass (%)	22.26 ± 2.19	25.28 ± 4.41	−2.884	0.016	−0.79 (−1.45 to −0.13)
FMI (kg·m^−2^)	5.06 ± 0.77	5.77 ± 1.45	−2.007	0.086	−0.56 (−1.21 to 0.10)
Skeletal muscle mass (kg)	27.25 ± 3.55	21.29 ± 3.07	5.399	<0.001	1.79 (1.04 to 2.55)
SMI (kg·m^−2^)	8.11 ± 0.69	7.28 ± 0.68	3.676	0.003	1.18 (0.49 to 1.88)
Muscle mass (kg)	30.68 ± 4.50	24.39 ± 4.03	4.448	0.001	1.46 (0.74 to 2.18)
Muscle-to-bone ratio	3.84 ± 0.44	3.56 ± 0.57	1.740	0.134	0.52 (−0.13 to 1.17)

Note: Values are presented as mean ± standard deviation (SD). Between-group differences were assessed using Welch’s *t*-test. *p*-values were adjusted using the Benjamini–Hochberg false discovery rate (FDR). Hedges’ g values are presented with 95% confidence intervals. FMI, fat mass index; SMI, skeletal muscle index.

**Table 3:** Somatotype values by playing position and competitive level.

Playing Position	Elite (Endo–Meso–Ecto)	Sub-Elite (Endo–Meso–Ecto)
Libero	2.9–4.2–2.7	3.9–4.5–1.7
Setter	3.1–3.9–2.8	4.5–4.1–1.9
Outside hitter	2.8–3.3–3.0	3.6–4.1–2.1
Opposite	3.6–2.8–3.6	3.3–2.9–3.1
Middle blocker	2.7–2.8–3.3	3.2–3.2–2.9

## Data Availability

The data supporting the findings of this study are not publicly available due to privacy and ethical restrictions. However, they can be accessed upon reasonable request from the corresponding author.

## Supplementary Materials

The following supporting information can be downloaded at https://www.mdpi.com/article/10.3390/sports14060223/s1: Supplementary Table S1. Descriptive statistics of volleyball players in the libero position by competition level (elite vs. sub-elite); Supplementary Table S2. Descriptive statistics of volleyball players in the setter position by competition level (elite vs. sub-elite); Supplementary Table S3. Descriptive statistics of volleyball players in the opposite position by competition level (elite vs. sub-elite); Supplementary Table S4. Descriptive statistics of volleyball players in the outside hitter position by competition level (elite vs. sub-elite); Supplementary Table S5. Descriptive statistics of volleyball players in the middle blocker position by competition level (elite vs. sub-elite).

## Abbreviations

The following abbreviations are used in this manuscript:

FM	Fat mass
FMI	Fat mass index
SMI	Skeletal muscle index
SMM	Skeletal muscle mass
