# Supplementary material for: Multilevel Analysis of Body Composition in Elite and Sub-Elite Female Volleyball Players: Structural and Potentially Modifiable Characteristics
Source: Sports (Basel). 2026 May 29;14(6):223. doi: 10.3390/sports14060223 (PMC13307310; doi:10.3390/sports14060223)
Supplement: Supplementary file 1 [file sports-14-00223-s001.zip › Supplementary Table S1_libero.pdf]

Supplementary Table S1. Descriptive statistics of volleyball players in the libero position by competition level (elite vs sub-elite).

Variable	Elite (n = 2)	Sub-elite (n = 4)
**General characteristics**		
Age (years)	31.50 ± 9.19	21.75 ± 6.85
Body mass (kg)	63.90 ± 2.26	60.70 ± 5.22
Stature (cm)	170.95 ± 2.90	162.43 ± 3.48
Sitting height (cm)	87.25 ± 1.06	88.42 ± 2.28
Arm span (cm)	166.15 ± 3.04	162.18 ± 1.20
BMI ($kg \cdot m^{-2}$)	21.89 ± 1.52	23.00 ± 1.81
Relative arm span (%)	97.22 ± 3.43	99.87 ± 1.43
Cormic index (%)	51.04 ± 0.25	54.44 ± 0.62
**Skinfold thicknesses**		
Triceps (mm)	11.00 ± 0.71	14.75 ± 3.38
Subscapular (mm)	9.00 ± 1.41	10.60 ± 2.70
Biceps (mm)	5.00 ± 1.41	6.67 ± 3.39
Iliac crest (mm)	14.75 ± 4.60	17.12 ± 7.25
Supraspinale (mm)	8.75 ± 1.06	10.85 ± 4.11
Abdominal (mm)	16.00 ± 5.66	17.50 ± 7.43
Thigh (mm)	22.00 ± 2.12	24.62 ± 7.17
Calf (mm)	10.50 ± 0.00	13.88 ± 6.32
Sum of 8 skinfolds (mm)	97.00 ± 14.85	116.00 ± 36.33
**Girths**		
Head (cm)	54.40 ± 1.56	54.97 ± 1.00
Neck (cm)	33.05 ± 0.21	32.08 ± 1.26
Arm relaxed (cm)	28.35 ± 2.76	27.95 ± 1.62
Arm flexed and tensed (cm)	29.20 ± 1.98	28.57 ± 1.86
Forearm (cm)	24.80 ± 0.57	23.77 ± 1.08
Wrist (cm)	15.65 ± 0.07	15.22 ± 0.83
Chest (cm)	89.85 ± 6.29	86.50 ± 4.87
Waist (cm)	72.25 ± 3.61	70.88 ± 3.70
Hip (cm)	97.00 ± 1.84	99.48 ± 5.61
Thigh 1 cm gluteal (cm)	59.75 ± 4.88	59.02 ± 2.65
Thigh (cm)	52.20 ± 2.26	51.88 ± 2.37
Calf (cm)	36.35 ± 1.06	35.40 ± 1.49
Ankle (cm)	23.40 ± 0.99	21.65 ± 0.78
**Lengths and proportional indices**		

Supplementary Table S1. Descriptive statistics of volleyball players in the libero position by competition level (elite vs sub-elite).

Variable	Elite (n = 2)	Sub-elite (n = 4)
Acromio-iliac index (%)	74.47 ± 1.86	73.14 ± 5.07
Acromiale–radiale (cm)	31.80 ± 1.98	30.38 ± 0.90
Radiale–stylion (cm)	25.00 ± 0.14	23.55 ± 0.85
Midstylion–dactylion (cm)	18.00 ± 0.00	17.77 ± 0.94
Iliospinale height (cm)	94.85 ± 1.20	90.95 ± 1.98
Trochanterion height (cm)	88.95 ± 0.64	84.70 ± 2.03
Trochanterion–tibiale laterale (cm)	44.10 ± 0.14	42.20 ± 1.10
Tibiale laterale height (cm)	44.65 ± 1.91	42.28 ± 0.75
Foot (cm)	24.60 ± 0.28	23.98 ± 1.98
Tibiale mediale–sphyrion tibiale (cm)	36.20 ± 3.11	36.30 ± 0.78
Brachial index (%)	78.78 ± 5.35	77.54 ± 2.36
Intermembral index (%)	78.88 ± 2.94	78.83 ± 1.47
Crural index (%)	82.10 ± 7.32	86.08 ± 3.36
**Breadths**		
Biacromial (cm)	38.00 ± 0.00	36.55 ± 1.16
Biiliocristal (cm)	28.30 ± 0.71	26.73 ± 1.94
Transverse chest (cm)	28.05 ± 0.07	26.08 ± 1.29
Antero-posterior chest (cm)	16.05 ± 1.48	16.55 ± 0.70
Antero-posterior abdominal depth (cm)	15.10 ± 3.54	18.28 ± 0.94
Humerus (cm)	6.40 ± 0.14	6.25 ± 0.37
Bi-styloid (cm)	5.30 ± 0.14	4.92 ± 0.47
Femur (cm)	9.45 ± 0.21	8.93 ± 0.32
Bimalleolar (cm)	6.75 ± 0.07	6.72 ± 0.32
**Ultrasound-derived variables**		
Biceps fat (cm)	0.18 ± 0.05	0.26 ± 0.11
Biceps muscle (cm)	2.75 ± 0.01	1.84 ± 0.34
Triceps fat (cm)	0.71 ± 0.02	0.59 ± 0.36
Abdominal fat (cm)	1.04 ± 0.59	1.12 ± 0.52
Abdominal muscle (cm)	1.08 ± 0.18	0.95 ± 0.12
Thigh fat (cm)	0.73 ± 0.00	0.88 ± 0.22
Thigh muscle (cm)	3.54 ± 0.20	4.33 ± 0.70
Calf fat (cm)	0.43 ± 0.01	0.53 ± 0.22
Calf muscle (cm)	1.60 ± 0.11	1.49 ± 0.39
Sum muscle thickness (cm)	8.97 ± 0.26	8.61 ± 1.30

Supplementary Table S1. Descriptive statistics of volleyball players in the libero position by competition level (elite vs sub-elite).

Variable	Elite (n = 2)	Sub-elite (n = 4)
Sum fat thickness (cm)	3.09 ± 0.64	3.37 ± 0.60
**Body mass components**		
Fat mass (kg)	14.33 ± 2.28	15.37 ± 1.53
Fat mass (%)	22.38 ± 2.78	25.35 ± 1.89
FMI ($kg \cdot m^{-2}$)	4.92 ± 0.95	5.84 ± 0.70
Skeletal muscle mass (kg)	21.97 ± 0.20	19.40 ± 2.40
SMI ($kg \cdot m^{-2}$)	7.52 ± 0.19	7.34 ± 0.74
Bone mass (kg)	7.42 ± 0.16	7.06 ± 0.63
Muscle mass (kg)	27.01 ± 2.87	23.60 ± 3.67
Muscle-to-bone ratio	3.64 ± 0.31	3.37 ± 0.64

Values are presented as mean ± standard deviation (SD). BMI = body mass index; FMI = fat mass index; SMI = skeletal muscle mass index.
