# Supplementary material for: Multilevel Analysis of Body Composition in Elite and Sub-Elite Female Volleyball Players: Structural and Potentially Modifiable Characteristics
Source: Sports (Basel). 2026 May 29;14(6):223. doi: 10.3390/sports14060223 (PMC13307310; doi:10.3390/sports14060223)
Supplement: Supplementary file 1 [file sports-14-00223-s001.zip › Supplementary Table S2_setter.pdf]

Supplementary Table S2. Descriptive statistics of volleyball players in the setter position by competition level (elite vs sub-elite).

Variable	Elite (n = 3)	Sub-elite (n = 5)
**General characteristics**		
Age (years)	24.33 ± 5.69	22.20 ± 7.16
Body mass (kg)	75.10 ± 5.81	67.64 ± 8.20
Stature (cm)	180.60 ± 4.67	169.50 ± 7.21
Sitting height (cm)	89.83 ± 3.12	88.90 ± 2.13
Arm span (cm)	182.50 ± 4.00	170.02 ± 10.16
BMI (kg·m^{-2})	23.03 ± 1.74	23.54 ± 2.43
Relative arm span (%)	101.08 ± 2.59	100.26 ± 2.61
Cormic index (%)	49.74 ± 0.95	52.49 ± 1.24
**Skinfold thicknesses**		
Triceps (mm)	13.83 ± 3.40	18.20 ± 5.63
Subscapular (mm)	9.83 ± 0.76	14.76 ± 6.68
Biceps (mm)	4.83 ± 0.58	9.24 ± 5.11
Iliac crest (mm)	13.50 ± 0.87	19.80 ± 11.30
Supraspinale (mm)	8.67 ± 2.47	11.52 ± 5.95
Abdominal (mm)	13.00 ± 2.18	22.08 ± 9.29
Thigh (mm)	19.00 ± 3.04	30.44 ± 5.58
Calf (mm)	13.50 ± 5.41	17.08 ± 4.13
Sum of 8 skinfolds (mm)	96.17 ± 12.57	143.12 ± 50.73
**Girths**		
Head (cm)	58.00 ± 2.86	54.60 ± 1.17
Neck (cm)	33.67 ± 0.74	33.88 ± 1.03
Arm relaxed (cm)	30.33 ± 1.88	29.38 ± 3.32
Arm flexed and tensed (cm)	32.37 ± 2.08	29.56 ± 2.60
Forearm (cm)	26.10 ± 0.75	24.76 ± 2.05
Wrist (cm)	16.10 ± 0.66	15.14 ± 0.80
Chest (cm)	92.00 ± 4.96	88.60 ± 5.19
Waist (cm)	77.50 ± 0.82	73.66 ± 7.47
Hip (cm)	104.57 ± 0.85	99.80 ± 3.90
Thigh 1 cm gluteal (cm)	60.83 ± 3.62	58.88 ± 3.41
Thigh (cm)	54.17 ± 5.51	51.64 ± 2.88
Calf (cm)	37.43 ± 0.95	36.56 ± 2.07
Ankle (cm)	22.63 ± 0.67	22.58 ± 1.42
**Lengths, heights, and proportional indices**		

Supplementary Table S2. Descriptive statistics of volleyball players in the setter position by competition level (elite vs sub-elite).

Variable	Elite (n = 3)	Sub-elite (n = 5)
Acromio-iliac index (%)	72.73 ± 7.04	75.28 ± 3.30
Acromiale–radiale (cm)	34.07 ± 1.70	32.22 ± 2.47
Radiale–stylion (cm)	26.73 ± 1.70	23.70 ± 1.32
Midstylion–dactylion (cm)	19.93 ± 0.25	18.34 ± 1.25
Iliospinale height (cm)	104.03 ± 5.34	96.42 ± 6.75
Trochanterion height (cm)	97.47 ± 2.06	89.84 ± 5.36
Trochanterion–tibiale laterale (cm)	48.43 ± 1.36	45.16 ± 2.97
Tibiale laterale height (cm)	49.73 ± 0.64	46.10 ± 2.89
Foot (cm)	26.53 ± 0.42	24.86 ± 1.27
Tibiale mediale–sphyrion tibiale (cm)	41.33 ± 2.75	39.50 ± 2.65
Brachial index (%)	78.52 ± 4.61	73.81 ± 5.67
Intermembral index (%)	77.66 ± 1.64	77.08 ± 1.68
Crural index (%)	85.30 ± 4.14	87.49 ± 3.01
**Breadths**		
Biacromial (cm)	39.67 ± 1.86	36.62 ± 2.84
Biiliocristal (cm)	28.77 ± 1.48	27.52 ± 1.72
Transverse chest (cm)	29.30 ± 1.44	26.80 ± 1.91
Antero-posterior chest (cm)	16.13 ± 0.50	16.72 ± 0.47
Antero-posterior abdominal depth (cm)	19.07 ± 1.37	18.16 ± 2.14
Humerus (cm)	6.80 ± 0.26	6.28 ± 0.36
Bi-styloid (cm)	5.37 ± 0.15	5.10 ± 0.31
Femur (cm)	9.27 ± 0.57	9.28 ± 0.23
Bimalleolar (cm)	7.10 ± 0.44	6.74 ± 0.49
**Ultrasound-derived variables**		
Biceps fat (cm)	0.31 ± 0.06	0.51 ± 0.21
Biceps muscle (cm)	2.62 ± 0.38	2.14 ± 0.44
Triceps fat (cm)	0.90 ± 0.26	1.23 ± 0.42
Abdominal fat (cm)	1.16 ± 0.60	1.44 ± 0.74
Abdominal muscle (cm)	1.39 ± 0.15	1.05 ± 0.22
Thigh fat (cm)	0.76 ± 0.12	1.07 ± 0.15
Thigh muscle (cm)	3.75 ± 0.47	3.67 ± 0.71
Calf fat (cm)	0.66 ± 0.22	0.77 ± 0.13
Calf muscle (cm)	1.63 ± 0.16	1.60 ± 0.11
Sum muscle thickness (cm)	9.39 ± 0.89	8.45 ± 1.01

Supplementary Table S2. Descriptive statistics of volleyball players in the setter position by competition level (elite vs sub-elite).

Variable	Elite (n = 3)	Sub-elite (n = 5)
Sum fat thickness (cm)	3.80 ± 0.66	5.02 ± 1.43
**Body mass components**		
Fat mass (kg)	17.82 ± 1.82	19.34 ± 4.80
Fat mass (%)	23.70 ± 0.59	28.34 ± 5.31
FMI ($kg \cdot m^{-2}$)	5.46 ± 0.52	6.76 ± 1.75
Skeletal muscle mass (kg)	26.18 ± 2.13	20.91 ± 2.92
SMI ($kg \cdot m^{-2}$)	8.03 ± 0.63	7.25 ± 0.60
Bone mass (kg)	7.81 ± 0.25	6.88 ± 1.18
Muscle mass (kg)	30.18 ± 2.64	24.08 ± 4.12
Muscle-to-bone ratio	3.86 ± 0.34	3.55 ± 0.60

Values are presented as mean ± standard deviation (SD). BMI = body mass index; FMI = fat mass index; SMI = skeletal muscle mass index;
