# Supplementary material for: Multilevel Analysis of Body Composition in Elite and Sub-Elite Female Volleyball Players: Structural and Potentially Modifiable Characteristics
Source: Sports (Basel). 2026 May 29;14(6):223. doi: 10.3390/sports14060223 (PMC13307310; doi:10.3390/sports14060223)
Supplement: Supplementary file 1 [file sports-14-00223-s001.zip › Supplementary Table S3_opposite.pdf]

Supplementary Table S3. Descriptive statistics of volleyball players in the opposite position by competition level (elite vs sub-elite).

Variable	Elite (n = 3)	Sub-elite (n = 6)
**General characteristics**		
Age (years)	24.33 ± 4.04	21.67 ± 6.80
Body mass (kg)	82.13 ± 4.23	68.07 ± 12.16
Stature (cm)	190.87 ± 10.68	176.57 ± 10.13
Sitting height (cm)	93.87 ± 3.33	91.85 ± 4.14
Arm span (cm)	195.17 ± 10.05	178.43 ± 8.58
BMI ($kg \cdot m^{-2}$)	22.62 ± 1.91	21.77 ± 2.97
Relative arm span (%)	102.29 ± 2.38	101.11 ± 1.66
Cormic index (%)	49.26 ± 2.72	52.05 ± 0.77
**Skinfold thicknesses**		
Triceps (mm)	16.00 ± 5.22	14.67 ± 4.36
Subscapular (mm)	13.67 ± 2.57	11.08 ± 4.51
Biceps (mm)	5.83 ± 2.52	5.80 ± 2.36
Iliac crest (mm)	18.50 ± 0.50	10.92 ± 4.12
Supraspinale (mm)	10.17 ± 1.76	7.78 ± 2.56
Abdominal (mm)	18.67 ± 5.86	15.32 ± 4.13
Thigh (mm)	22.67 ± 6.11	22.50 ± 9.51
Calf (mm)	10.50 ± 2.00	15.00 ± 5.29
Sum of 8 skinfolds (mm)	116.00 ± 17.20	103.07 ± 33.83
**Girths**		
Head (cm)	56.77 ± 1.86	55.48 ± 1.84
Neck (cm)	35.30 ± 2.95	33.32 ± 1.62
Arm relaxed (cm)	29.97 ± 1.01	27.90 ± 2.77
Arm flexed and tensed (cm)	31.17 ± 1.17	28.38 ± 2.14
Forearm (cm)	26.57 ± 0.75	24.37 ± 1.58
Wrist (cm)	16.87 ± 0.32	15.72 ± 0.80
Chest (cm)	97.53 ± 8.67	90.37 ± 7.31
Waist (cm)	78.67 ± 3.37	72.67 ± 6.62
Hip (cm)	107.10 ± 2.59	100.45 ± 6.46
Thigh 1 cm gluteal (cm)	62.07 ± 1.97	58.05 ± 5.28
Thigh (cm)	53.40 ± 2.55	49.50 ± 3.92
Calf (cm)	37.67 ± 2.61	35.20 ± 3.29
Ankle (cm)	24.27 ± 0.81	21.87 ± 1.81
**Lengths, heights, and proportional indices**		

Supplementary Table S3. Descriptive statistics of volleyball players in the opposite position by competition level (elite vs sub-elite).

Variable	Elite (n = 3)	Sub-elite (n = 6)
Acromio-iliac index (%)	73.64 ± 3.16	75.20 ± 5.01
Acromiale–radiale (cm)	37.33 ± 1.70	32.37 ± 2.88
Radiale–stylion (cm)	28.37 ± 1.99	26.32 ± 1.67
Midstylion–dactylion (cm)	21.73 ± 0.59	18.77 ± 1.19
Iliospinale height (cm)	111.73 ± 9.22	100.15 ± 5.86
Trochanterion height (cm)	104.10 ± 10.12	94.18 ± 5.69
Trochanterion–tibiale laterale (cm)	52.57 ± 4.19	48.00 ± 2.92
Tibiale laterale height (cm)	52.37 ± 5.39	46.55 ± 2.94
Foot (cm)	27.87 ± 1.06	25.32 ± 1.58
Tibiale mediale–sphyrion tibiale (cm)	42.03 ± 6.53	41.00 ± 2.58
Brachial index (%)	75.97 ± 3.67	81.91 ± 10.08
Intermembral index (%)	78.42 ± 3.15	77.38 ± 2.48
Crural index (%)	79.95 ± 11.05	85.43 ± 2.11
**Breadths**		
Biacromial (cm)	41.30 ± 2.42	37.43 ± 2.27
Biiliocristal (cm)	30.37 ± 0.90	28.17 ± 2.73
Transverse chest (cm)	29.63 ± 2.01	26.63 ± 1.71
Antero-posterior chest (cm)	17.40 ± 1.21	16.12 ± 1.05
Antero-posterior abdominal depth (cm)	18.53 ± 0.90	19.03 ± 1.84
Humerus (cm)	6.97 ± 0.15	6.48 ± 0.40
Bi-styloid (cm)	5.73 ± 0.06	5.20 ± 0.24
Femur (cm)	9.77 ± 0.25	9.13 ± 0.62
Bimalleolar (cm)	7.30 ± 0.10	6.88 ± 0.39
**Ultrasound-derived variables**		
Biceps fat (cm)	0.30 ± 0.17	0.29 ± 0.17
Biceps muscle (cm)	2.90 ± 1.36	1.97 ± 0.24
Triceps fat (cm)	0.78 ± 0.20	0.93 ± 0.43
Abdominal fat (cm)	1.33 ± 0.77	1.32 ± 0.68
Abdominal muscle (cm)	1.43 ± 0.20	1.14 ± 0.10
Thigh fat (cm)	0.93 ± 0.26	0.78 ± 0.29
Thigh muscle (cm)	3.82 ± 0.60	3.26 ± 0.65
Calf fat (cm)	0.50 ± 0.21	0.68 ± 0.30
Calf muscle (cm)	1.83 ± 0.16	1.47 ± 0.06
Sum muscle thickness (cm)	9.98 ± 1.73	7.84 ± 0.79

Supplementary Table S3. Descriptive statistics of volleyball players in the opposite position by competition level (elite vs sub-elite).

Variable	Elite (n = 3)	Sub-elite (n = 6)
Sum fat thickness (cm)	3.84 ± 1.09	4.00 ± 1.76
**Body mass components**		
Fat mass (kg)	17.92 ± 1.84	17.30 ± 6.86
Fat mass (%)	21.88 ± 2.87	24.65 ± 5.76
FMI ($kg{\cdot}m^{-2}$)	4.93 ± 0.55	5.49 ± 1.91
Skeletal muscle mass (kg)	30.21 ± 2.99	21.75 ± 2.33
SMI ($kg{\cdot}m^{-2}$)	8.32 ± 0.97	7.00 ± 0.80
Bone mass (kg)	8.13 ± 0.81	6.87 ± 1.01
Muscle mass (kg)	31.62 ± 4.51	24.74 ± 4.04
Muscle-to-bone ratio	3.89 ± 0.36	3.62 ± 0.40

Values are presented as mean ± standard deviation (SD). BMI = body mass index; FMI = fat mass index; SMI = skeletal muscle mass index.
