# Supplementary material for: Multilevel Analysis of Body Composition in Elite and Sub-Elite Female Volleyball Players: Structural and Potentially Modifiable Characteristics
Source: Sports (Basel). 2026 May 29;14(6):223. doi: 10.3390/sports14060223 (PMC13307310; doi:10.3390/sports14060223)
Supplement: Supplementary file 1 [file sports-14-00223-s001.zip › Supplementary Table S4_outside hitter.pdf]

Supplementary Table S4. Descriptive statistics of volleyball players in the outside hitter position by competition level (elite vs sub-elite).

Variable	Elite (n = 5)	Sub-elite (n = 5)
**General characteristics**		
Age (years)	24.00 ± 3.54	22.00 ± 7.04
Body mass (kg)	74.66 ± 6.45	66.34 ± 10.55
Stature (cm)	181.88 ± 3.10	169.76 ± 5.79
Sitting height (cm)	87.72 ± 2.56	88.64 ± 2.96
Arm span (cm)	188.62 ± 5.69	172.98 ± 5.68
BMI ($kg \cdot m^{-2}$)	22.60 ± 2.26	22.90 ± 2.09
Relative arm span (%)	103.71 ± 2.74	101.92 ± 2.17
Cormic index (%)	48.25 ± 1.96	52.23 ± 1.27
**Skinfold thicknesses**		
Triceps (mm)	12.70 ± 4.93	15.74 ± 2.44
Subscapular (mm)	9.30 ± 0.57	10.46 ± 1.87
Biceps (mm)	5.40 ± 1.14	6.56 ± 2.05
Iliac crest (mm)	11.70 ± 3.09	17.72 ± 1.29
Supraspinale (mm)	7.30 ± 2.08	8.96 ± 1.05
Abdominal (mm)	12.90 ± 3.23	19.14 ± 2.40
Thigh (mm)	17.60 ± 5.64	26.52 ± 7.59
Calf (mm)	8.50 ± 2.45	14.98 ± 2.58
Sum of 8 skinfolds (mm)	85.40 ± 15.34	120.08 ± 14.37
**Girths**		
Head (cm)	57.10 ± 2.68	53.48 ± 1.58
Neck (cm)	33.40 ± 1.32	33.08 ± 1.81
Arm relaxed (cm)	29.10 ± 1.74	28.22 ± 3.45
Arm flexed and tensed (cm)	30.86 ± 2.07	28.70 ± 2.85
Forearm (cm)	25.94 ± 0.76	24.40 ± 1.74
Wrist (cm)	16.14 ± 0.54	15.60 ± 0.81
Chest (cm)	92.92 ± 5.59	88.14 ± 4.38
Waist (cm)	76.16 ± 4.85	72.86 ± 5.20
Hip (cm)	100.58 ± 3.98	99.38 ± 5.18
Thigh 1 cm gluteal (cm)	61.16 ± 2.79	59.32 ± 5.11
Thigh (cm)	54.96 ± 2.57	53.36 ± 5.34
Calf (cm)	35.72 ± 2.37	36.68 ± 2.48
Ankle (cm)	22.64 ± 1.28	22.08 ± 0.98
**Lengths, heights, and proportional indices**		

Supplementary Table S4. Descriptive statistics of volleyball players in the outside hitter position by competition level (elite vs sub-elite).

Variable	Elite (n = 5)	Sub-elite (n = 5)
Acromio-iliac index (%)	72.99 ± 3.24	77.98 ± 13.55
Acromiale–radiale (cm)	35.22 ± 1.44	32.58 ± 1.38
Radiale–stylion (cm)	28.20 ± 1.37	24.54 ± 1.15
Midstylion–dactylion (cm)	20.30 ± 1.02	18.92 ± 1.19
Iliospinale height (cm)	105.62 ± 4.65	96.96 ± 4.78
Trochanterion height (cm)	102.02 ± 5.35	94.00 ± 4.02
Trochanterion–tibiale laterale (cm)	52.10 ± 3.45	44.66 ± 3.46
Tibiale laterale height (cm)	51.12 ± 2.98	45.34 ± 2.14
Foot (cm)	27.10 ± 0.98	25.20 ± 1.38
Tibiale mediale–sphyrion tibiale (cm)	43.40 ± 3.42	38.92 ± 1.70
Brachial index (%)	80.11 ± 3.45	75.35 ± 2.82
Intermembral index (%)	79.32 ± 1.90	78.51 ± 3.40
Crural index (%)	83.37 ± 5.26	87.37 ± 4.24
**Breadths**		
Biacromial (cm)	39.68 ± 1.43	35.44 ± 4.55
Biiliocristal (cm)	28.94 ± 1.07	27.18 ± 1.49
Transverse chest (cm)	28.68 ± 1.33	25.82 ± 1.47
Antero-posterior chest (cm)	16.52 ± 0.68	16.26 ± 0.93
Antero-posterior abdominal depth (cm)	18.20 ± 1.13	19.00 ± 1.66
Humerus (cm)	6.66 ± 0.15	6.44 ± 0.36
Bi-styloid (cm)	5.36 ± 0.23	5.12 ± 0.28
Femur (cm)	9.60 ± 0.23	9.32 ± 0.41
Bimalleolar (cm)	7.18 ± 0.29	6.86 ± 0.43
**Ultrasound-derived variables**		
Biceps fat (cm)	0.36 ± 0.15	0.34 ± 0.09
Biceps muscle (cm)	2.33 ± 0.23	2.08 ± 0.35
Triceps fat (cm)	0.67 ± 0.13	0.99 ± 0.19
Abdominal fat (cm)	0.85 ± 0.34	1.04 ± 0.26
Abdominal muscle (cm)	1.40 ± 0.23	1.12 ± 0.21
Thigh fat (cm)	0.67 ± 0.24	0.93 ± 0.23
Thigh muscle (cm)	4.18 ± 0.43	3.79 ± 0.30
Calf fat (cm)	0.38 ± 0.16	0.63 ± 0.13
Calf muscle (cm)	1.62 ± 0.28	1.61 ± 0.24
Sum muscle thickness (cm)	9.52 ± 0.56	8.60 ± 0.72

Supplementary Table S4. Descriptive statistics of volleyball players in the outside hitter position by competition level (elite vs sub-elite).

Variable	Elite (n = 5)	Sub-elite (n = 5)
Sum fat thickness (cm)	2.92 ± 0.62	3.93 ± 0.75
**Body mass components**		
Fat mass (kg)	16.16 ± 3.29	16.94 ± 4.00
Fat mass (%)	21.48 ± 2.80	25.31 ± 1.92
FMI ($kg{\cdot}m^{-2}$)	4.90 ± 1.07	5.83 ± 0.98
Skeletal muscle mass (kg)	26.99 ± 1.80	21.68 ± 3.50
SMI ($kg{\cdot}m^{-2}$)	8.16 ± 0.60	7.48 ± 0.69
Bone mass (kg)	7.74 ± 0.44	6.45 ± 1.33
Muscle mass (kg)	30.61 ± 4.35	25.42 ± 5.71
Muscle-to-bone ratio	3.97 ± 0.66	3.97 ± 0.59

Values are presented as mean ± standard deviation (SD). BMI = body mass index; FMI = fat mass index; SMI = skeletal muscle mass index.
