# Supplementary material for: Multilevel Analysis of Body Composition in Elite and Sub-Elite Female Volleyball Players: Structural and Potentially Modifiable Characteristics
Source: Sports (Basel). 2026 May 29;14(6):223. doi: 10.3390/sports14060223 (PMC13307310; doi:10.3390/sports14060223)
Supplement: Supplementary file 1 [file sports-14-00223-s001.zip › Supplementary Table S5_middle blocker.pdf]

Supplementary Table S5. Descriptive statistics of volleyball players in the middle blocker position by competition level (elite vs sub-elite).

Variable	Elite (n = 2)	Sub-elite (n = 5)
**General characteristics**		
Age (years)	23.50 ± 0.71	22.40 ± 6.11
Body mass (kg)	83.30 ± 14.85	64.76 ± 8.59
Stature (cm)	190.20 ± 3.25	172.52 ± 10.60
Sitting height (cm)	94.95 ± 0.07	92.36 ± 4.11
Arm span (cm)	197.75 ± 4.60	181.22 ± 11.72
BMI ($kg \cdot m^{-2}$)	22.97 ± 3.32	21.73 ± 1.82
Relative arm span (%)	103.96 ± 0.64	105.21 ± 7.25
Cormic index (%)	49.93 ± 0.82	53.61 ± 2.40
**Skinfold thicknesses**		
Triceps (mm)	11.00 ± 1.41	13.90 ± 3.94
Subscapular (mm)	10.00 ± 0.71	9.70 ± 3.29
Biceps (mm)	5.75 ± 1.77	5.48 ± 2.23
Iliac crest (mm)	11.75 ± 0.35	11.60 ± 3.49
Supraspinale (mm)	8.50 ± 0.00	8.46 ± 2.70
Abdominal (mm)	16.00 ± 1.41	14.62 ± 4.41
Thigh (mm)	19.00 ± 4.24	18.06 ± 6.12
Calf (mm)	13.50 ± 4.95	9.94 ± 4.40
Sum of 8 skinfolds (mm)	95.50 ± 14.85	91.76 ± 26.54
**Girths**		
Head (cm)	56.40 ± 3.68	56.24 ± 1.62
Neck (cm)	34.80 ± 0.71	33.22 ± 2.28
Arm relaxed (cm)	29.75 ± 1.77	26.72 ± 2.15
Arm flexed and tensed (cm)	30.15 ± 2.19	27.52 ± 2.09
Forearm (cm)	26.15 ± 0.78	23.52 ± 1.64
Wrist (cm)	16.10 ± 0.71	15.74 ± 0.51
Chest (cm)	96.60 ± 9.19	87.36 ± 3.96
Waist (cm)	76.10 ± 8.34	71.24 ± 1.00
Hip (cm)	108.75 ± 7.99	95.42 ± 6.76
Thigh 1 cm gluteal (cm)	64.10 ± 5.52	55.56 ± 3.35
Thigh (cm)	55.10 ± 4.53	48.98 ± 4.03
Calf (cm)	39.15 ± 3.61	34.56 ± 1.67
Ankle (cm)	25.65 ± 1.63	21.24 ± 0.99
**Lengths, heights, and proportional indices**		

Supplementary Table S5. Descriptive statistics of volleyball players in the middle blocker position by competition level (elite vs sub-elite).

Variable	Elite (n = 2)	Sub-elite (n = 5)
Acromio-iliac index (%)	71.06 ± 1.53	73.57 ± 4.34
Acromiale–radiale (cm)	38.05 ± 0.49	32.36 ± 6.42
Radiale–stylion (cm)	28.65 ± 0.21	26.98 ± 0.94
Midstylion–dactylion (cm)	22.05 ± 0.21	19.12 ± 0.93
Iliospinale height (cm)	107.00 ± 6.36	100.44 ± 4.25
Trochanterion height (cm)	103.95 ± 7.57	94.04 ± 4.26
Trochanterion–tibiale laterale (cm)	51.00 ± 3.39	46.98 ± 2.21
Tibiale laterale height (cm)	51.95 ± 1.20	47.34 ± 2.20
Foot (cm)	27.45 ± 1.48	25.36 ± 1.59
Tibiale mediale–sphyrion tibiale (cm)	40.70 ± 0.71	41.08 ± 1.62
Brachial index (%)	75.31 ± 1.54	83.37 ± 1.03
Intermembral index (%)	83.08 ± 4.48	78.02 ± 5.30
Crural index (%)	79.93 ± 3.93	87.48 ± 1.48
**Breadths**		
Biacromial (cm)	44.15 ± 3.04	38.28 ± 2.02
Biiliocristal (cm)	31.35 ± 1.48	28.20 ± 2.83
Transverse chest (cm)	29.30 ± 2.69	27.18 ± 1.10
Antero-posterior chest (cm)	15.00 ± 0.71	14.76 ± 0.70
Antero-posterior abdominal depth (cm)	16.50 ± 0.00	17.88 ± 1.07
Humerus (cm)	6.75 ± 0.07	6.40 ± 0.42
Bi-styloid (cm)	5.55 ± 0.21	5.30 ± 0.22
Femur (cm)	9.85 ± 0.64	9.20 ± 0.29
Bimalleolar (cm)	7.15 ± 0.35	7.02 ± 0.25
**Ultrasound-derived variables**		
Biceps fat (cm)	0.34 ± 0.05	0.25 ± 0.17
Biceps muscle (cm)	2.56 ± 0.25	1.97 ± 0.14
Triceps fat (cm)	0.87 ± 0.18	0.80 ± 0.34
Abdominal fat (cm)	1.11 ± 0.15	0.79 ± 0.49
Abdominal muscle (cm)	1.32 ± 0.05	1.02 ± 0.21
Thigh fat (cm)	0.70 ± 0.13	0.61 ± 0.16
Thigh muscle (cm)	4.27 ± 0.58	3.65 ± 0.79
Calf fat (cm)	0.54 ± 0.19	0.38 ± 0.18
Calf muscle (cm)	1.71 ± 0.12	1.35 ± 0.17
Sum muscle thickness (cm)	9.87 ± 1.00	7.98 ± 1.13

Supplementary Table S5. Descriptive statistics of volleyball players in the middle blocker position by competition level (elite vs sub-elite).

Variable	Elite (n = 2)	Sub-elite (n = 5)
Sum fat thickness (cm)	3.55 ± 0.70	2.84 ± 1.13
**Body mass estimations**		
Fat mass (kg)	18.80 ± 4.26	14.68 ± 2.72
Fat mass (%)	22.47 ± 1.11	22.89 ± 4.61
FMI ($kg{\cdot}m^{-2}$)	5.18 ± 1.00	4.99 ± 1.26
Skeletal muscle mass (kg)	30.30 ± 5.68	22.23 ± 4.40
SMI ($kg{\cdot}m^{-2}$)	8.35 ± 1.28	7.41 ± 0.72
Bone mass (kg)	9.32 ± 2.04	7.47 ± 1.10
Muscle mass (kg)	33.86 ± 9.69	23.89 ± 3.84
Muscle-to-bone ratio	3.61 ± 0.25	3.24 ± 0.61

Values are presented as mean ± standard deviation (SD). BMI = body mass index; FMI = fat mass index; SMI = skeletal muscle mass index.
